# Cochrane corner: rapid point-of-care antigen and molecular-based tests for the diagnosis of COVID-19 infection

**DOI:** 10.11604/pamj.supp.2020.37.1.25982

**Published:** 2020-09-15

**Authors:** Duduzile Ndwandwe, Lindi Mathebula, Raoul Kamadjeu, Charles Shey Wiysonge

**Affiliations:** 1 Cochrane South Africa, South African Medical Research Council, Cape Town, South Africa,; 2 Communicable Disease Control, Department of Health, Western Cape, South Africa,; 3 UNICEF Program Division, Public Health Emergencies Team, New York, USA,; 4 School of Public Health and Family Medicine, Faculty of Health Sciences, University of Cape Town, South Africa,; 5 Department of Global Health, Faculty of Medicine and Health Sciences, Stellenbosch University, Cape Town, South Africa

**Keywords:** COVID-19, rapid point of care test, SARS-CoV-2 diagnostic strategies

## Abstract

**Introduction:**

the COVID-19 pandemic, which results from infection with severe acute respiratory syndrome coronavirus 2 (SARS-CoV-2), presents important diagnostic challenges. Diagnostic strategies available to identify or rule out current infection, or to identify people in need of care escalation, or to test for past infection and immune response have become available, to reduce household and community transmission. We highlight a Cochrane review, published in September 2020, on the assessment of diagnostic accuracy of point-of-care antigen and molecular-based tests to determine current SARS-CoV-2 infection.

**Methods:**

the authors of the Cochrane review searched multiple electronic databases for studies, which assessed SARS-CoV-2 infection with a diagnostic test. Eligible participants for the review included people with suspected current SARS-CoV-2 infection, known to have, or not to have COVID-19 infection, or where tests were used to screen for infection.

**Results:**

the authors included 18 studies of point-of-care tests conducted in various parts of the world, with none from Africa. The review shows that there is considerable variability in sensitivity and specificity of the antigen tests. The review also shows that molecular tests had less variability in sensitivity and specificity.

**Conclusion:**

the review suggests that the current evidence is not strong enough to determine the usefulness of point-of-care tests in all settings. However, the benefits are likely to be more noticeable in countries, like Africa where community transmission is high. An impact evaluation would be warranted when rapid point-of-care tests are implemented in African countries.

## Introduction

The diagnosis of COVID-19 is based on a combination of clinical symptoms with or without radiological imaging, confirmed by SARS-CoV-2 PCR [1]. Severe acute respiratory syndrome coronavirus 2 (SARS-CoV-2) and the resulting COVID-19 pandemic present important diagnostic challenges [2]. Several diagnostic strategies are available to identify current infection, rule out infection, identify people in need of care escalation, or to test for past infection and immune response [3]. Several bottlenecks have been reported such as the time-consuming process with sample ribonucleic acid (RNA) extraction and polymerase chain reaction (PCR) run-time of approximately 6 hours and a turnaround time of 12-24 hours. Providing fast results is part of the WHO´s testing and tracing backbone strategy for COVID-19 response. This approach of critical importance in a time of shortage of medical personnel, protective materials and beds on isolation wards and to ensure timely and adequate treatment for patients, developing high-quality rapid point of care diagnostics is essential. Antigenic tests have the potential of providing a quick diagnosis. In this commentary, we discuss a Cochrane review of rapid, point-of-care antigen and molecular-based tests for diagnosis of SARS-CoV-2 infection [2]. The review by Dinnes and colleagues assessed the rapid, point-of-care antigen and molecular-based tests for diagnosis of SARS-CoV-2 infection.

## Methods

The review assessed the diagnostic accuracy of point-of-care antigen and molecular-based tests used to determine if a person presenting in the community or primary or secondary care has current SARS-CoV-2 infection [4-6]. On the 25 May 2020, the authors conducted electronic searches in the Cochrane COVID-19 Study Register, COVID-19 Living Evidence Database from the University of Bern, and repositories of COVID-19 publications checked with no language restrictions. The primary consideration for the eligibility of tests for inclusion in this review was that the studies should detect current infection and should have the capacity to be performed at the 'point of care' or in a 'near-patient' testing role. Studies were screened, data extracted, and risk of bias assessed in duplicate. Data were presented for sensitivity and specificity using a paired forest plot. Antigen and molecular-based tests were also presented separately.

## Results

The review included 22 publications reporting 18 study cohorts with 3198 unique samples, of which 1775 had confirmed SARS-CoV-2 infection. The studies were conducted in North America, South America, Europe, and China. One study was conducted in multiple countries. The authors identified data for eight commercial tests (four antigen and four molecular) and one in-house antigen test. There was no included study that had a low risk of bias for all quality domains. Risk of bias refers to systematic errors in the way the studies were conducted, analysed or interpreted. For example, test review bias occurs when results of the reference standard are known while the index test is interpreted. Patient selection in 50% of the included studies was found to have a high risk of bias resulting from over-representation of samples with confirmed COVID-19 infection. The risk of bias in seven studies was unclear because of poor reporting. Sixteen studies used only a single, negative reverse transcriptase polymerase chain reaction (RT-PCR) to confirm the absence of COVID-19 infection. There was a lack of information on blinding of the index test in 11 studies and a lack of information around participant exclusions from the analyses in 10 studies. However, the authors did not observe differences in methodological quality between antigen and molecular test evaluations. The sensitivity of antigen tests varied considerably across studies, from 0% to 94% in eight evaluations of 943 samples. The average sensitivity was 56.2% with 95% confidence interval (CI) 29.5% to 79.8%, and the average specificity was 99.5% (95% CI 98.1% to 99.9%). The rapid molecular tests displayed less variation in sensitivity, ranging from 68% to 100% in 13 evaluations of 2255 samples. The average sensitivity of rapid molecular assays was 95.2% (95% CI 86.7% to 98.3%) and specificity 98.9% (95% CI 97.3% to 99.5%). For the most commonly used individual tests, the review found the sensitivity for the Xpert Xpress assay to be 99.4% (95% CI 98.0% to 99.8%) and that of the ID NOW assay to 76.8% (95% CI 72.9% to 80.3%). The respective specificities for the two tests were 96.8% (95% CI 90.6% to 99.0%) and 99.6% (95% CI 98.4% to 99.9%.

## Discussion

The review reiterates the importance of rapid diagnosis of SARS-CoV-2 infection in curbing the spread of the disease. Reliable and early detection of cases is a critical backbone of COVID-19 response strategy [2,7]. The benefits of point of care rapid testing have been demonstrated in several countries, however, the majority of African countries lack resources to procure some of these diagnostic tests on a wide scale [7]. Areas with widespread community transmission of SARS-CoV-2 and detection by RT-PCR of a single discriminatory target is considered sufficient. However, specific technical considerations for laboratory testing, including specimen collection (variable collection methods), which samples to collect (upper or lower respiratory tract biospecimens, or other samples), time of collection in relation to the course of disease, and the availability of different laboratory test methods and kits [8].

Notably, most of the studies included in this review were conducted in middle to higher-income countries with a few studies conducted in multiple countries. While the results of the included studies in this review indicate that rapid, point of care testing can be used, the authors expressed concerns around application of evidence to other settings due to lack of reporting in some of the studies with most of the studies having a considerably high risk of bias. The authors also indicated that point of care testing using these diagnostic tests would be better suited in low prevalence areas. Findings presented in this review further show that point of care diagnostic tests often exhibit variable specificity and sensitivity levels. African countries have a high burden of SARS-CoV-2 infections and the testing rollout is mostly dependent on each country´s preferred COVID-19 control strategy and sometimes prevailing circumstances [7]. According to WHO, several diagnostic features can be used in testing strategies for SARS-CoV-2, however, suspected cases should be validated by laboratory tests [7]. Currently, RT-PCR detection of unique sequences of the viral genome is the gold standard for COVID-19 [7]. It is noted that RT-PCR is labour-intensive and takes longer for results to be available. Rapid diagnostic tests with a short turnaround time would be useful in ensuring immediate diagnosis of SARS-CoV-2 to limit the spread of SARS-CoV-2 and for the clinical management of COVID-19 in Africa where community transmission of SARS-CoV-2 is prevalent [9].

## Conclusion

The findings from this review are likely to have different implications for policy and practices in African countries. Rapid diagnostic tests with a short turnaround time would be useful in ensuring immediate diagnosis of SARS-CoV-2 to limit the spread of SARS-CoV-2 and for the clinical management of COVID-19 in Africa where community transmission of SARS-CoV-2 is prevalent. More studies should be conducted in low- and middle- income countries specifically for the context of Africa to evaluate the suitability of such rapid, point of care diagnostic test.

### What is known about this topic

- Rapid, point of care diagnosis have been developed to incorporate as part of screening strategies for COVID-19;
- Extensive validation across different populations is required before the tests can be routinely used to inform critical decision making for clinicians, the public health community and policy makers;
- No systematic review on the sensitivity and specificity of these tests published before the Cochrane review.

### What this study adds

- Point of care diagnosis for SARS-CoV-2 has a potential to be a useful tool in screening protocols to rapid respond to the evolving pandemic;
- The benefits of point of care diagnosis may be more pronounced in countries with community transmission.
